# Microstructures and Soft Magnetic Properties of Fe_73.5−x_Cu_1_Nb_3_Si_13.5_B_9_Gd_x_ (x = 0–1.5) Alloys

**DOI:** 10.3390/ma15092973

**Published:** 2022-04-19

**Authors:** Yuchen Mao, Zhenghou Zhu, Hui Zhao

**Affiliations:** 1 College of Materials Science and Engineering, Nanchang University, Nanchang 330031, China; maoyuchen87@126.com; 2 Dayou Technology Co., Ltd., Yichun 336000, China; 3 Institute of Space Science and Technology, Nanchang University, Nanchang 330031, China

**Keywords:** Fe-based nanocrystalline alloy, soft magnetic properties, rare earth Gd

## Abstract

In this experiment, the rare earth Gd element was added to Finemet alloy to observe the microstructure and soft magnetic properties. The experimental results showed that the samples with the addition of 0.5% Gd and 1.0% Gd can be quenched and cast normally, and the *M_S_* of Fe_73_Cu_1_Nb_3_Si_13.5_B_9_Gd_0.5_ alloy was 10.41% higher than that of Finemet. After annealing, crystal grains of about 10 nm were formed. The *μ_i_* and *μ_m_* values of Fe_73_Cu_1_Nb_3_Si_13.5_B_9_Gd_0.5_ alloy were 25.51% and 22.23% higher, respectively, and the coercivity *H_C_* was reduced by 12.19% compared to Finemet. At 1 kHz, the *μ_e_* value of Fe_73_Cu_1_Nb_3_Si_13.5_B_9_Gd_0.5_ alloy at room temperature was 14.57% higher than that of Finemet, while the *μ_e_* reached 162.34 k and 142.42 k at 90 °C and 150 °C (24% and 29.51% higher, respectively). The Fe_72.5_Cu_1_Nb_3_Si_13.5_B_9_Gd_1.0_ alloy had the best performance at 100 kHz, with higher *μ_e_* values than Finemet across the ambient temperature range of 30 °C to 150 °C. After tension annealing, the *μ_e_* values of Fe_72.5_Cu_1_Nb_3_Si_13.5_B_9_Gd_1.0_ alloy were 20–30% higher than those of Finemet.

## 1. Introduction

Using ultrafast cooling technology to cool the liquid metal to room temperature, and to form a solid alloy with a disordered arrangement of atoms, amorphous samples can be obtained. Amorphous alloys have received much attention since their inception; their intrinsic quality [1] and glass formation [2] have been explored. Amorphous materials exhibit special properties due to the disordered arrangement of atoms, e.g., strength, hardness, and corrosion resistance [3,4].

Soft magnetic materials have excellent magnetic properties and usability, and they are widely used in high-tech fields such as aviation and aerospace. With the development of human society and technology, the requirements for lightweight and miniaturized soft magnetic material equipment are increasing. Therefore, the development of excellent soft magnetic materials with high saturation magnetic induction, high permeability, low coercivity, and low iron loss has become one of the important goals of current and future research [5].

Amorphous soft magnetic materials emerged in the late 1970s. Due to the long-range disorder of atoms, the anisotropy of the material is greatly reduced, which is beneficial to obtain high permeability and low coercivity. As a soft magnetic material with excellent performance, iron-based amorphous alloys can be used in various electronic and power components [6,7,8], such as switching power supplies [9], transformers, transducers, filters [10], and sensors. Due to the low no-load loss, transformers with iron-based amorphous alloys have been widely used in rural power grids with long no-load time and low power efficiency, which has significant energy-saving effects. Due to the excellent corrosion resistance, iron-based amorphous alloys can be applied to new and powerful sensors to meet the needs of new technology fields such as automobiles, biological robots, power motors, chemicals, and medical electronics. In addition, due to the high initial magnetic permeability, iron-based amorphous alloys can be used to develop various devices for high-sensitivity occasions to ensure precision and stability [11].

Although iron-based amorphous alloys have high saturation magnetic induction and low coercivity, the effective permeability at the high frequencies is much lower than that of cobalt-based amorphous alloys, which limits the application of iron-based amorphous alloys in high-frequency magnetic components. Due to the high cost of Co raw materials, people have been trying to improve the high-frequency permeability of iron-based amorphous alloys to replace cobalt-based amorphous alloys in some special applications. In 1988, the Finemet alloy developed by Yoshizawa from Hitachi Metals exhibited both high saturation magnetic flux density and effective magnetic permeability, which was a breakthrough in the research of soft magnetic materials [12]. On the basis of this research, a series of nanocrystalline soft magnetic materials were developed, with the more famous ones being Nanoperm [13] and Hitperm [14] alloys. Nanocrystalline materials require 10–15 nm crystal grains dispersed in the amorphous matrix; hence, there is a process of nucleation and growth during the annealing process. The atomic nucleation mechanism and retention of nanocrystalline phases in materials have important effects on the properties [15,16,17].

In the production of the steel industry, rare earth elements have been widely used as impurity scavengers in steel melting and casting processes. Because the reactions between rare earth elements and harmful impurities such as oxygen or sulfur are thermodynamically more favored compared to those between impurities and Fe [18], a small addition can achieve good results; accordingly, it has been called “the vitamin of modern industry”. Adding a small amount of rare earth elements to steel has the functions of removing, deoxidizing, and refining grains. Rare earth elements have magnetism because their internal *f*-electron shells are not completely filled. Therefore, the addition of small amounts of magnetic rare earth elements will affect not only the GFA (glass-forming ability) of bulk glass-forming alloys, but also their magnetic properties.

The magnetic properties of rare earth elements mainly originate from the *f* orbitals which are not filled. From a microscopic point of view, the magnetism is not due to the coupling of the cooperative exchange phenomenon (direct exchange or super exchange); it is because of the coupling between the orbital momentum and the spin of each atom. The rare earth element gadolinium (atomic number 64) has a special outer electron arrangement, with one electron in each of the seven orbitals of the 4*f* sublayer, which means that it has the largest number of unpaired electrons among all rare earth elements and, thus, the highest “natural” spin value. In this way, gadolinium has the largest magnetic moment of the unpaired electron and has a special spin dynamic mechanism [19,20]. Gadolinium has a wide range of applications in alloys.

During the formation of amorphous alloys, intermetallic compounds can be regarded as competing phases of the amorphous phase. The acquisition of the amorphous alloy depends on the mutual interference and hindrance between the competing phases [21]. Gd element has stronger affinity toward oxygen, i.e., Gd and oxygen react preferentially in thermodynamics. Oxygen can significantly reduce the formation ability of amorphous alloys; thus, in molten liquid alloy, a small amount of Gd element also plays the role of oxygen adsorbent during the amorphous formation process, which suppresses the adverse effects of oxygen in the process of melting and casting, suppresses heterogeneous nucleation, and improves the ability of amorphous formation [22,23].

With the development of smart grids, the requirements for the accuracy of the upgraded smart meter transformer cores have increased. The main requirement is that the *μ_e_* remains constant over a wide frequency range and wide external fields. The traditional process is to use transverse magnetic annealing to reduce the low-frequency *μ_e_* value of Finemet, and the material can maintain the stability of the *μ_e_* value in a wide frequency range. However, transverse magnetic annealing is limited by the size of the external magnetic field of the equipment, and tension annealing is more effective than transverse magnetic annealing [24]. In this experiment, a sample with rare earth element added was treated by constant tension annealing, and the change in its magnetic properties was tested.

In this paper, Gd element was added to the Finemet alloy; processes in actual industrial production were selected to prepare the magnetic cores, and the structures and magnetic properties were compared with the Finemet alloy without Gd.

## 2. Experimental Procedures

Using the processes of vacuum melting and rapid cooling, Fe_73.5−x_Cu_1_Nb_3_Si_13.5_B_9_Gd_x_ (x = 0, 0.5, 1.0, and 1.5) amorphous alloy ribbons were prepared, and their formulas are shown in Table 1.

In this experiment, the alloy Fe_73.5−x_Cu_1_Nb_3_Si_13.5_B_9_Gd_x_ (x = 0, 0.5, and 1.0) was made into amorphous ribbons with a thickness of 24–26 μm, while the Fe_72_Cu_1_Nb_3_Si_13.5_B_9_Gd_1.5_ alloy had poor fluidity, and only a small amount of ribbons were prepared, without magnetic cores.

Since the samples of this experiment were prepared on a small scale, in order to facilitate the comparison with existing products, the annealing treatment was processed together with mass-produced products. Therefore, an existing annealing process and magnetic core size were selected. The amorphous alloy ribbons were wound into toroidal cores of Φ25 mm (outer diameter) × Φ16 mm (inner diameter) × 10 mm (height). Six samples of each formula were produced, and they were annealed in a vacuum furnace at 525 °C or 557 °C, with three samples for each annealing temperature. After annealing, the corresponding plastic shells were selected for packing. In addition, part of the as-cast Fe_72.5_Cu_1_Nb_3_Si_13.5_B_9_Gd_1_ alloy and original Finemet alloy ribbons were subjected to constant tension annealing at 600 °C and 630 °C, respectively, using self-made equipment from Dayou-Tech Company (Yichun, Jiangxi, China). After tension annealing, all ribbons were wound into Φ18.3 mm (outer diameter) × Φ12.8 mm (inner diameter) × 10 mm (height) toroidal magnetic cores and packaged into corresponding plastic shells.

The static soft magnetic performances were tested using an MATS-2010SD soft magnetic DC measuring device (*Hi* = 0.08 A/m, *Hj* = 0.8 A/m, *Hs* = 40 A/m, N1:N2 = 5:2); a Tonghui Electronics (Changzhou, Hunan, China) TH2829C precision LCR meter was used to test the inductance values at different temperatures and frequencies. In the magnetic performance test results obtained, the average value of the three samples was taken as the final performance value.

A Bruker D8 Advance XRD (X-ray diffractometer, Berlin, Germany) was used to analyze the crystallization (CuKα, 40 kV, 30 mA, 2*θ* = 20–90°, 8°/min); an NETZSCH differential thermal analyzer (Bavaria, Germany) was used to measure differential thermal data (heating rate 10 °C/min, Ar protection, gas flow 100 mL/min); a Lakeshore 7410 VSM (vibrating sample magnetometer, Westerville, USA) was used to test coercivity and magnetization values. A tungsten filament scanning electron microscope from Hitachi (Tokyo, Japan) was used to observe microstructure, and EDS (energy-dispersive spectroscopy) was used to analyze the element contents. The instrument used to observe microscopic morphology was a Tecnai G2 F30 (FEI Company, Hillsboro, OL, USA) field-emission TEM (transmission electron microscope) operated at 300 kV; 3DAP (CAMECA Instruments LEAP 5000XR, Madison, WI, USA) was used to detect the content and distribution of each element.

## 3. Results and Analysis

### 3.1. Microstructures

The samples annealed at 557 °C were selected for microstructure observation.

**Figure 1:**
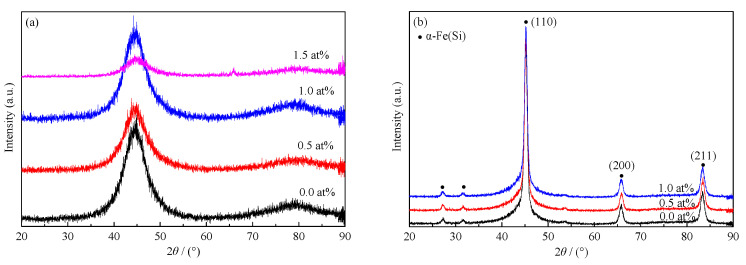
X-ray diffraction data before and after annealing. (**a**): as-casted alloy; (**b**): annealed alloy.

The X-ray diffraction test results in Figure 1 show that all as-cast alloys were amorphous. During the casting process, the fluidity of the 1.5% Gd alloy was poor, and there was a certain segregation of components due to the addition of a large amount of Gd, which led to the uneven structure of the alloy melt and affected the casting process. During the annealing process, the amorphous alloys began to crystallize, and the solid solution phase precipitated. Compared with the PDF card (ICDD PDF 2004), the three strongest peaks (45.2°, 67.4°, and 84.2°) and small peaks of 27° and 32° all corresponded to the α-Fe(Si) phase (PDF: No. 35-0519). With the increase in annealing temperature, the precipitation of α-Fe(Si) phase was more significant; Fe_73.5−x_Cu_1_Nb_3_Si_13.5_B_9_Gd_x_ (x = 0, 0.5, and 1.0) samples were normally precipitated in α-Fe(Si) solid solution phase. The estimation of the crystal phase by Jade software is shown in Table 2.

With Gd addition, the interplanar distance or grain size changed little, which proves that the addition of Gd had little effect.

**Figure 2:**
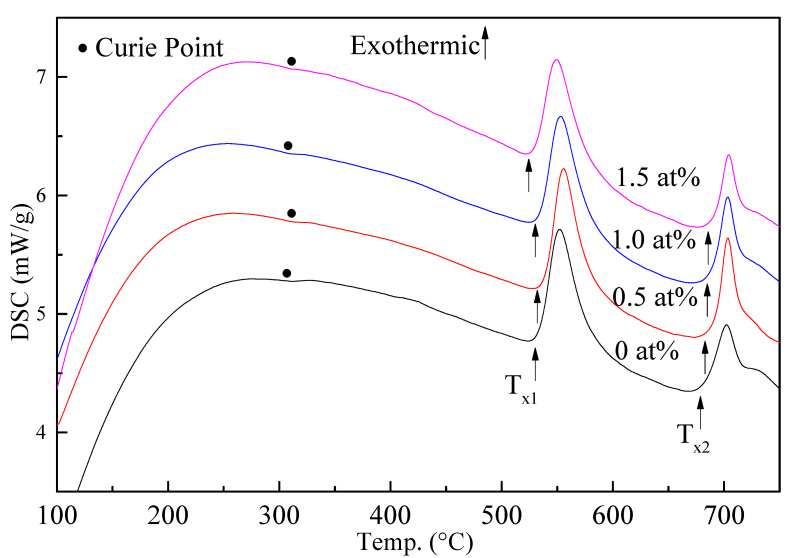
DSC data of as-cast alloys.

In Figure 2, we can see two distinct crystallization peaks. The iron–silicon phase was precipitated in the first crystallization peak, and the FeB phase was precipitated in the second crystallization peak. During the annealing process, it is desirable to obtain a nanosized iron–silicon phase without precipitation of the FeB phase. Compared with the alloy without Gd, the first and second crystallization peaks of the 0.5% sample were slightly shifted to a higher-temperature region, indicating that the amorphous structure of the as-cast alloy was more stable, and the crystallization treatment needed a higher annealing temperature. When Gd content increased to 1.0% and 1.5%, the first crystallization peak of the alloy moved to a lower-temperature region, while the second crystallization peak continued to move to a higher-temperature region, and the distance between the two crystallization peaks was wider, indicating that the annealing processing temperature could be broader.

**Figure 3:**
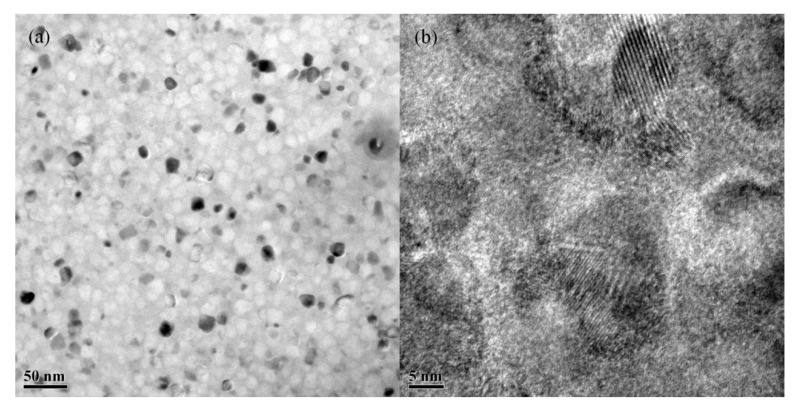
TEM morphology of 1.0% Gd sample. (**a**,**b**) for different magnifications.

Obvious crystal grains can be seen in Figure 3, roughly spherical in shape. The grain size was relatively as seen in Figure 3a, with no abnormally coarse grains. In this experiment, the addition of Gd element reduced the Fe content, while the Nb and Cu contents, which have a greater effect on the grain size, remained unchanged, thus proving that the addition of Gd had no significant effect on the grain size.

As shown in Figure 3b, the grain size was about 10 nm, similar to the XRD results, indicating that the sample would have good soft magnetic properties [25]. Fine crystal planes can be seen, and the crystal composition was in the iron–silicon phase.

**Figure 4:**
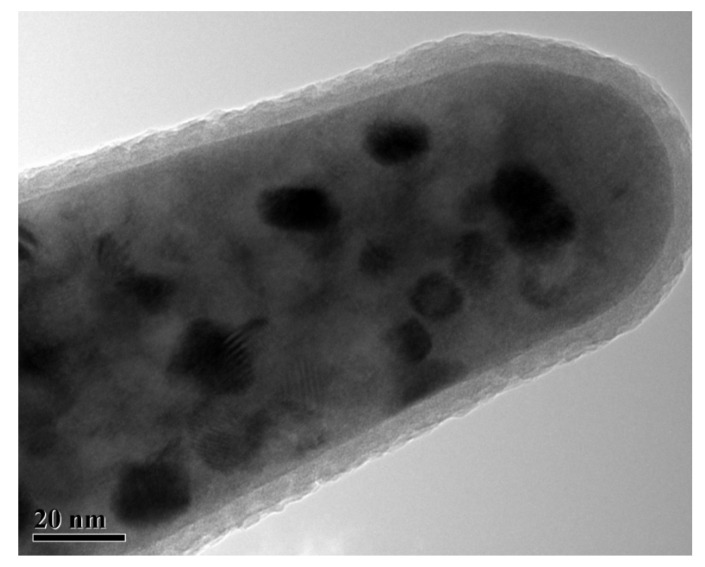
TEM morphology of APT sample.

The APT (atom probe test) allows determining the element distribution. However, the APT test completely consumes the sample; thus, the sample structure was observed by TEM before APT. The obvious crystal grain shape can be seen in Figure 4, with the outer surface layer consisting of an amorphous region formed by FIB (focused ion beam).

**Figure 5:**
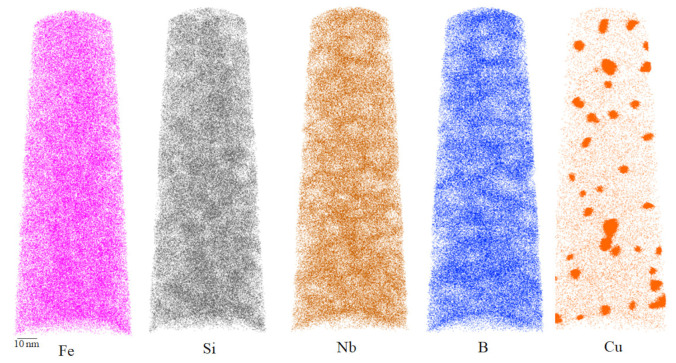
Atom probe analysis of 1.0% sample.

In Figure 5, we can see the distribution of each element. Unfortunately, the Gd element was not found. Since the atom probe only takes a region with a width of about 60 nm and a length of about 150–200 nm, if there is component segregation in the sample, the Gd region may not be detected. In addition, it is also possible that, during the smelting process, the Gd element reacted with impurities such as oxygen and sulfur, and was taken away during the slag removal process.

The distribution of other elements was obvious. Copper was present in clusters, with a size of 4–6 nm, which could serve as nucleation points for the iron–silicon phase [26]. Iron and silicon elements formed the iron–silicon phase, with a similar distribution in the grains. Niobium and boron elements were mainly distributed at the grain boundaries.

**Figure 6:**
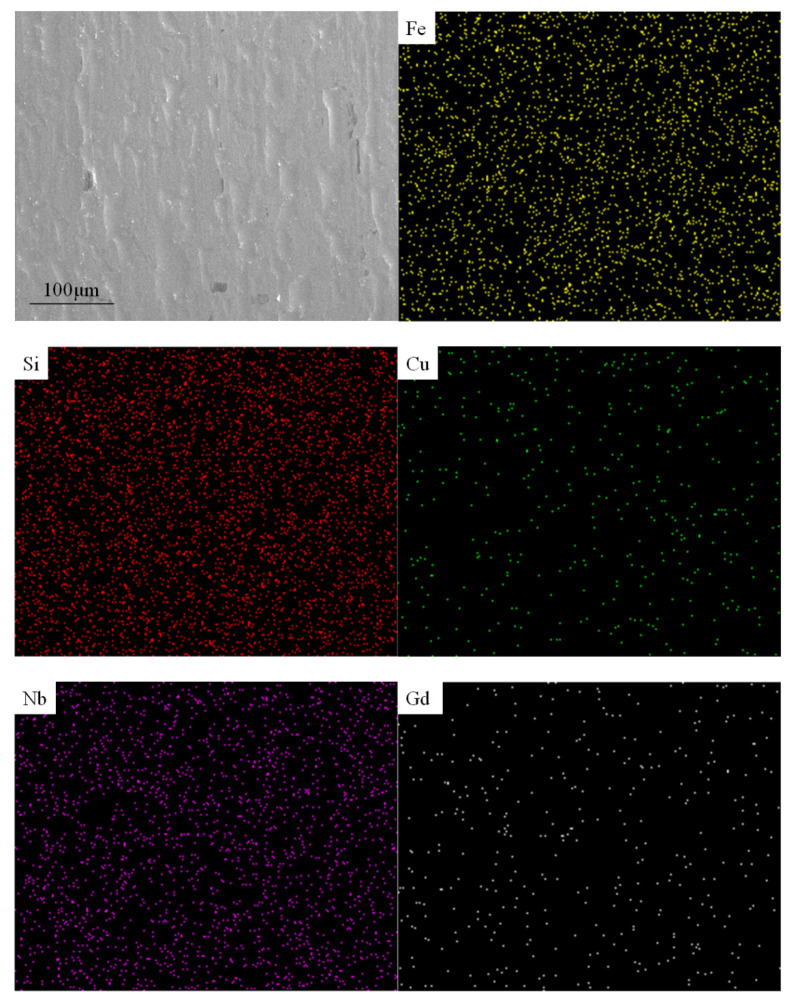

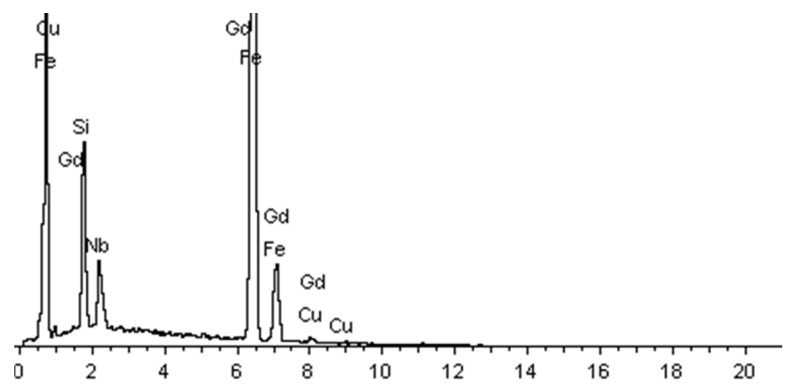
SEM and EDS of 1.0% Gd sample.

EDS was used to scan a larger area to confirm the incorporation of the Gd element into the matrix. As shown in Figure 6, no obvious distribution can be seen from the EDS spectrum, but it can be confirmed that the alloy sample did contain Gd element. The content of other elements is shown in Table 3.

It can be seen that the content of the Gd element was much lower than the actual addition amount. This indicates that most of the Gd element was consumed during the smelting and casting process.

### 3.2. Soft Magnetic Properties

#### 3.2.1. Static Soft Magnetic Performance

It can be seen from Table 4 that, compared with Finemet, the samples with 0.5% Gd and 1.0% Gd had lower *H_C_* values and higher *M_S_* values, indicating that the addition of Gd could improve the overall soft magnetic properties of as-cast alloys. *H_C_* of the 1.0% sample was 17.92% lower than that of Finemet, while *M_S_* of the 0.5% sample was 10.41% higher.

Table 5 shows that the 0.5% Gd sample had better soft magnetic properties than Finemet. The *μ_i_* and *μ_m_* values were 25.51% and 22.23% higher, respectively. The *H_C_* value of the 0.5% Gd sample was reduced by 12.19% compared with Finemet, close to the test data of VSM, in which the *H_C_* of the 0.5% Gd sample was 13.40% lower than that of Finemet.

The *B_S_* value of the 0.5% Gd sample was slightly higher than that of Finemet. Theoretically, as the Gd element increases and the iron element decreases, the *B_S_* value should decrease. However, Finemet is a type of dual-phase material, and the change in the phase structure will also affect the *B_S_* value. From the DSC data of the 0.5% Gd sample (Figure 2), the Curie temperature and the crystallization peak were shifted to a higher-temperature region, indicating that the Gd element preferentially reacts with oxygen and sulfur, thus playing a role in removing impurities during the alloy melting process. This increases the alloy’s purity, reduces the consumption of iron, and improves the *B_S_* value.

As shown in Table 6, all samples annealed at 525 °C had better *μ_m_* and *B_S_* values; for the 1.0% sample, a lower annealing temperature was required for *B_r_* and a higher annealing temperature was required for *H_C_*.

#### 3.2.2. Dynamic Soft Magnetic Performance

In terms of dynamic magnetic properties *μ_e_*, the samples annealed at 557 °C had better performance; hence, the test results of all samples annealed at 557 °C were selected.

Table 7 shows that the 0.5% Gd sample had the best performance at 1 kHz, 14.57% higher than Finemet; the 1.0% Gd sample had the best performance at 10 kHz and 100 kHz, slightly higher than Finemet (3.98% and 5.23% higher, respectively).

#### 3.2.3. Performances at High Temperature and Different Frequencies

**Figure 7:**
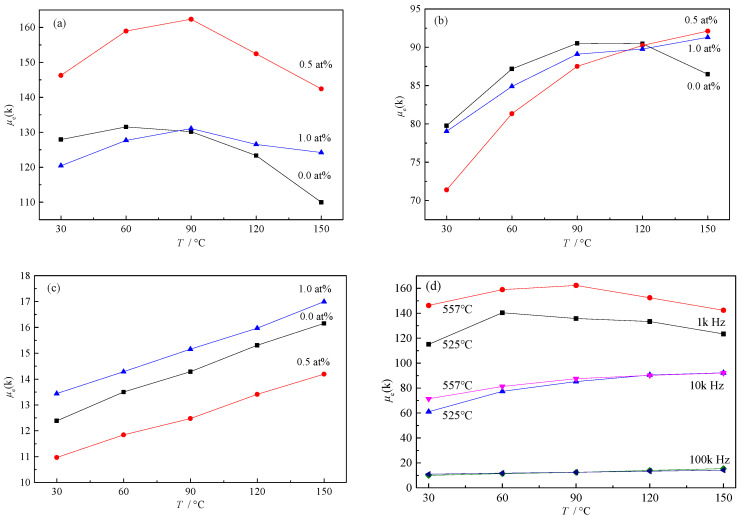
Evolution of μe values in the temperature range of 30–150 °C: (**a**–**c**) μe values at 1 kHz, 10 kHz, and 100 kHz, respectively (annealed at 557 °C); (**d**) performance comparison of 0.5% Gd alloys annealed at different temperatures.

As can be seen in Figure 7, the performance of the 0.5% Gd sample was significantly improved over the temperature range of 30 °C to 150 °C at a frequency of 1 kHz. The *μ_e_* of the 0.5% Gd sample reached 162.34 k and 142.42 k at 90 °C and 150 °C, respectively, 24% and 29.51% higher than Finemet. When the frequency increased to 10 kHz, Finemet alloy had the best performance below 120 °C; however, as the temperature increased above 120 °C, the performances of the 0.5% and 1.0% Gd samples, as well as Finemet, increased. At 150 °C, the samples with 0.5% and 1.0% Gd outperformed Finemet, indicating that the samples with added Gd had better performance at high temperature. According to the 100 kHz data, the overall performance of the 1.0% Gd sample was better, indicating that the samples with Gd addition also had better performance at high frequencies.

In addition, for the 0.5% Gd samples, the performance gap between the two heat treatment processes was small, proving that the 0.5% Gd sample could achieve excellent performance in a wide annealing temperature range. Thus, it can be applied in industrial production. Since the annealing process in production is carried out in large quantities, there are areas of uneven temperature in the annealing furnace. The stable annealing performance of the 0.5% Gd sample over a wide temperature range means that the temperature control does not need to be as stringent, which reduces the production process cost.

#### 3.2.4. Soft Magnetic Performance after Tension Annealing

The 0.0% and 1.0% Gd as-cast samples were selected for tension annealing, with annealing temperatures of 600 °C and 630 °C. The static magnetic properties are shown in Table 8.

The samples with Gd added had lower *μ* and *B* values than Finemet, as well as higher *H_C_*. Therefore, according to the static soft magnetic performance data, the addition of Gd is detrimental to the material. However, this type of product mainly relies on its effective permeability, and the most important parameter is the stability of the dynamic soft magnetic performance *μ_e_* value at different frequencies.

**Figure 8:**
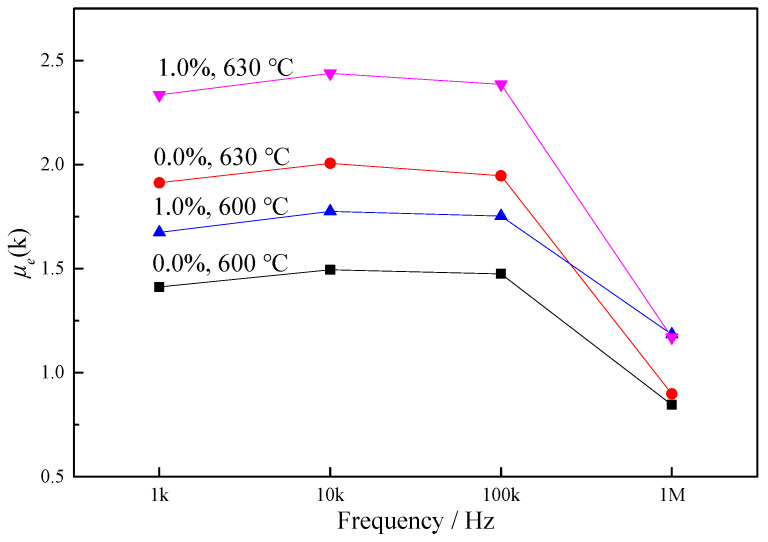
Dynamic soft magnetic performance after tension annealing.

At low frequency, the *μ_e_* value is mainly affected by the magnetic domain deflection; at high frequency, the soft magnetic performance is mainly affected by eddy current loss. As can be seen from the high-frequency performance in Figure 8, compared with ordinary annealing, the *μ_e_* value of the samples after tensile annealing was much lower. The *μ_e_* value of the samples annealed at 630 °C was higher compared to 600 °C (same results as obtained for the static magnetic properties). The *μ_e_* value dropped significantly when the frequency was increased to 1 MHz. Currently, the application range of Finemet products is mainly below 1 MHz; hence, an annealing temperature of 630 °C is more suitable. After annealing at 630 °C, the *μ_e_* value of the 1.0% Gd sample was 20–30% higher than that of Finemet at all frequencies, which shows that the addition of Gd could improve the dynamic magnetic properties, and that Finemet with Gd addition would be more suitable for the tension annealing process.

Although this product mainly requires stable magnetic permeability at different frequencies, if the magnetic permeability value can be increased in a stable manner, the product volume, the number of copper coils, and the overall device cost can be reduced in practical applications.

**Figure 9:**
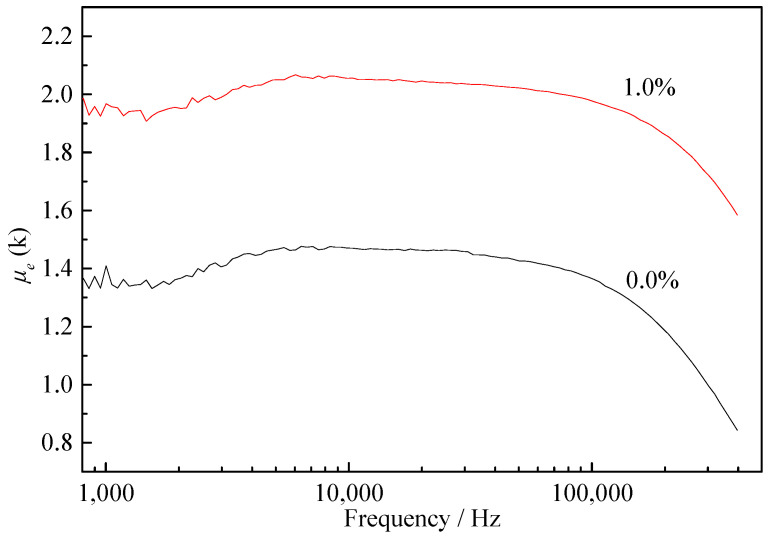
Comparison of frequency curves of 0.0% alloy and 1.0% alloy.

As shown in Figure 9, the *μ_e_* value decreased with the increase in frequency. Before 100 kHz, the *μ_e_* value changed very little, whereas, after 100 kHz, the *μ_e_* value began to decrease rapidly. According to the *μ_e_* value at 100 kHz, the frequency point at which the *μ_e_* value of the Gd-free alloy decreased by 10% was 175.54 kHz, while that for the 1.0% Gd alloy was 256.85 kHz, indicating a slower decline trend of the 1.0% Gd sample. Therefore, the 1.0% Gd alloy had better frequency stability and could adapt to wider frequency requirements.

## 4. Conclusions

(1) During the casting process, the Fe_72_Cu_1_Nb_3_Si_13.5_B_9_Gd_1.5_ alloy exhibited poor fluidity during smelting, and it could not be quenched and cast into ribbons. Therefore, in practical applications, the amount of Gd addition should be within 1.0%. After annealing, the Fe_72.5_Cu_1_Nb_3_Si_13.5_B_9_Gd_1.0_ alloy had uniformly fine grains, with a grain size of about 10 nm. The Gd element removed impurities during the preparation process, and most of the Gd was consumed.
(2) Compared with Finemet, the samples with Gd addition had lower coercive and higher saturation magnetization. The coercivity of the Fe_72.5_Cu_1_Nb_3_Si_13.5_B_9_Gd_1.0_ alloy was 17.92% lower, and the saturation magnetization of the Fe_73_Cu_1_Nb_3_Si_13.5_B_9_Gd_0.5_ alloy was 10.41% higher. The overall magnetic properties of the alloy were all better than those of Finemet. The *μ_i_* and *μ_m_* values were 25.51% and 22.23% higher compared to Finemet, respectively, while the coercivity *H_C_* was reduced by 12.19%.
(3) At room temperature, Fe_73_Cu_1_Nb_3_Si_13.5_B_9_Gd_0.5_ alloy had the best performance at 1 kHz, with a 14.57% higher *μ_e_* than Finemet; Fe_72.5_Cu_1_Nb_3_Si_13.5_B_9_Gd_1.0_ alloy had the best performance at 10 kHz and 100 kHz, 3.98% and 5.23% higher than Finemet, respectively. In the temperature range of 30 °C to 150 °C, when the frequency was 1 kHz, the *μ_e_* value of Fe_72.5_Cu_1_Nb_3_Si_13.5_B_9_Gd_1.0_ alloy was significantly improved and reached 162.34 k at 90 °C and 142.42 k at 150 °C, 24% and 29.51% higher than Finemet. At 10 kHz, the sample with Gd added outperformed Finemet at 150 °C; at 100 kHz, the Fe_72.5_Cu_1_Nb_3_Si_13.5_B_9_Gd_1.0_ alloy performed best. In addition, after the tension annealing, the *μ_e_* value of the Fe_72.5_Cu_1_Nb_3_Si_13.5_B_9_Gd_1.0_ alloy was 20–30% higher compared to Finemet at all frequencies, indicating that Finemet with Gd addition is more suitable for the tensile annealing process.

Unfortunately, in this experiment, we did not determine the location or distribution of Gd element. Hence, in the future, more Gd can be added with traditional casting to observe the distribution of Gd in the grain boundary structure.

## Figures and Tables

**Table 1:** The numbers of moles of all components.

Gd at.%	Molecular Formulas of All Components
0.0%	Fe_73.5_Cu_1_Nb_3_Si_13.5_B_9_
0.5%	Fe_73_Cu_1_Nb_3_Si_13.5_B_9_Gd_0.5_
1.0%	Fe_72.5_Cu_1_Nb_3_Si_13.5_B_9_Gd_1_
1.5%	Fe_72_Cu_1_Nb_3_Si_13.5_B_9_Gd_1.5_

**Table 2:** Crystallization peaks of samples.

Gd at.%	2*θ*	Interplanar Distance (nm)	Grain Size (nm)
0.0%	45.217	0.20037	10.4
0.5%	45.259	0.20019	10.6
1.0%	45.238	0.20028	10.3

**Table 3:** Element contents of 1.0% samples.

Element	wt.%	at.%
Si	8.01	15.18
Fe	83.52	79.64
Cu	1.30	1.09
Nb	7.06	4.05
Gd	0.12	0.04

**Table 4:** Magnetic properties measured from VSM (as-cast alloys).

Gd at.%	Coercivity (*H_C_*)	Magnetization (*M_S_*)
0.0%	1.06 Oe	134.32 emu/g
0.5%	0.91 Oe	148.30 emu/g
1.0%	0.87 Oe	135.91 emu/g

**Table 5:** Static soft magnetic performances.

Gd at.%	*μ_i_* (k)	*μ_m_* (k)	*B_S_* (mT)	*B_r_* (mT)	*H_C_* (A/m)
0.0%	118.26	881.75	1200.00	774.62	0.41
0.5%	148.43	1077.75	1221.25	793.20	0.36
1.0%	122.44	643.80	1162.75	771.60	0.66

**Table 6:** Annealing temperatures for optimal static soft magnetic properties (°C).

Gd at.%	*μ_i_* (k)	*μ_m_* (k)	*B_S_* (mT)	*B_r_* (mT)	*H_C_* (A/m)
0.0%	557	525	525	557	525
0.5%	525	525	525	557	525
1.0%	557	525	525	525	557

**Table 7:** Dynamic magnetic performances of *μ_e_* value.

Gd at.%	1 kHz	10 kHz	100 kHz
0.0%	108.39	76.38	12.81
0.5%	124.18	69.33	10.88
1.0%	120.33	79.42	13.48

**Table 8:** Static soft magnetic performances after tension annealing.

Gd at.%	Temp. (°C)	*μ_i_* (k)	*μ_m_* (k)	*B_S_* (mT)	*B_r_* (mT)	*H_C_* (A/m)
0.0%	600	3.74	102.32	1126.25	426.23	2.73
1.0%	600	2.42	31.33	1035.50	307.48	5.03
0.0%	630	7.12	108.98	1164.00	410.30	2.10
1.0%	630	3.15	45.99	1134.40	318.66	4.05

## Data Availability

Exclude this statement if the study did not report any data.
